# St John’s wort use in Australian general practice patients with depressive symptoms: their characteristics and use of other health services

**DOI:** 10.1186/1472-6882-14-204

**Published:** 2014-06-26

**Authors:** Marie Pirotta, Konstancja Densley, Kirsty Forsdike, Meg Carter, Jane Gunn

**Affiliations:** 1 Department of General Practice, University of Melbourne, 200 Berkeley Street, Carlton 3053Victoria, Australia

**Keywords:** St John’s wort, *Hypericum perforatum*, Depression, General practice, Complementary therapies, Patient care

## Abstract

**Background:**

While depression is frequently managed by general practitioners, often patients self-manage these symptoms with alternative therapies, including St John’s wort (SJW). We tested whether use of SJW was associated with different patterns of conventional and complementary health service use, strategies used for management of depression, or user dissatisfaction with or lack of trust in their general practitioner or clinic overall.

**Methods:**

Secondary analysis of data collected from an Australian population screened for a longitudinal cohort study of depression. Main outcome measures were CES-D for depressive symptoms, satisfaction with their general practitioner (GPAQ), Trust in Physician scale, self-report of health services usage and strategies used to manage depression, stress or worries.

**Results:**

Response rate was 7667/17,780 (43.1%). Of these, 4.3% (320/7,432) had used SJW in the past 12 months (recent ‘SJW users’). SJW users were significantly more likely to be depressed and to have a higher CES-D score. There were no statistically significant differences between recent SJW users and non-SJW users in satisfaction with their general practice or in trust in their general practitioner (GP) when adjusted for multiple factors. SJW users were significantly more likely to use all health services, whether conventional or complementary, as well as other strategies used for mental health care. SJW users were also more likely to consider themselves the main carer for their depression.

**Conclusions:**

Primary care attendees with symptoms of depression who use SJW appear not to be rejecting conventional medicine. Rather, they may be proactive care seekers who try both conventional and complementary strategies to manage their depressive symptoms. If GPs enquire and find that their depressed patients are using SJW, this may indicate that they might explore for unrelieved symptoms of depression and also consider the issue of potential for interactions between SJW and other medicines.

## Background

Depression is a condition commonly managed in general practice. Treatments available to people experiencing depression and other mental health problems include prescription medicines, over-the-counter treatments, care from medical and other practitioners, as well as self-initiated options [1]. Many people with mental health issues do not seek formal care [2].

St John’s wort (SJW, botanical name *Hypericum perforatum*) has been used for centuries in the treatment of mental health disorders. SJW is a herbal medicine that doctors need to be aware of due to its popularity, evidence of its effectiveness in depression, and its potential for interactions with other medicines. SJW forms part of the standard care for depression in Germany [3] and has been found to be one of the more popular complementary and alternative medicines (CAM) used by consumers in the US [4]. In an Australian community-based sample of three age cohorts (N = 7485), over the past month 5.6% had taken antidepressant medicines and 1.1% had used a complementary or alternative medicine (CAM) for depression and 2.4% for anxiety, of which, two-thirds (63%) had taken SJW [5].

Of all CAM, SJW has been the most tested in clinical trials [6]. A Cochrane review found evidence to support the use of SJW for treatment of major depression [7]. Although SJW has fewer side effects than conventional antidepressants, it has clinically important interactions with some conventional drugs, in particular, with selective serotonin reuptake inhibitors (SSRIs) [3]. It is for this reason that the National Institute for Health and Care Excellence (NICE) clinical guidelines do not recommend its use in the United Kingdom.

Herbal medicines are regulated slightly differently in different countries, which may have an impact on its availability to consumers. SJW is regulated in the UK by the Medicines Healthcare products Regulatory Agency (MHRA). To sell a product containing SJW over-the-counter in the UK, companies must obtain either full Marketing Authorisation or Traditional Herbal Registration (THR). The former requires evidence of efficacy, but companies can choose to follow the route of THR which only requires evidence of traditional use, safety, and quality of the product. In Australia, SJW is regulated by the Therapeutic Goods Administration as a listed medicine, in a similar process to THR. SJW may be recommended to patients by their general practitioners [8] or by complementary therapists; however, SJW can also be purchased over-the-counter with no professional input, which is a concern due to its potential for interactions with other medicines.

Little research has addressed why consumers use SJW [2]. An interview-based study conducted in the United States with 22 current users of SJW found four dominant decision-making themes: (1) participants (or their parents) had a history of CAM use and believed in personal control of their heath; (2) lowered mood; (3) self-diagnosis of “minor” depression, high risks of prescription drugs, and a perception of safety with herbal remedies, and (4) barriers to and lack of knowledge of conventional health care providers and awareness of the ease of use and popularity of SJW [9].

No research has explored how people who use SJW for depressive symptoms also use other treatments, whether conventional or complementary or alternative. We explored whether primary care attendees, who had used SJW to manage depressive symptoms in the last 12 months, were also more likely to use other strategies and components of the conventional and complementary health care systems to manage their depressive symptoms, and whether they differed from non-SJW users in their satisfaction with or trust in their general practitioner and their clinic overall. Our hypothesis was that recent use of SJW would not be associated with marked differences in health service use or in attitudes to general practice compared with people who had not used SJW.

## Methods

We undertook a secondary cross sectional analysis of screening data from a large cohort study in general practice, which explores how patients deal with depression, stress or worries called the Diagnosis, Management and Outcomes of Depression in Primary Care (*diamond*) study conducted in Victoria, Australia. *diamond* is a prospective, longitudinal cohort study of general practice attendees with depression from 30 randomly selected metropolitan and rural general practices, that began in January 2005. Clinic records were searched to identify all people aged 18-75 years who had seen the study GP in the previous year. Each GP examined this list and excluded those people who could not read English, were terminally ill, or resided in a nursing home. Between January and December 2005, random samples of about 600 eligible people from each general practice were mailed a screening survey with a covering letter from the GP and one follow up reminder letter after two weeks.

In addition to this initial survey, we report some results from a baseline telephone interview, conducted on average four weeks after the survey, between January 2005 and April 2006, with participants who met defined diagnostic criteria for depression. The study received Human Research Ethics Committee Approval from The University of Melbourne and full details of the methods have been reported [10].

### Participant sample

Of the 17,780 people initially sent a screening survey as part of the *diamond* study, 7,667 (43%) returned a completed survey. The mean age of people who were sent the screening survey was 46.2 years (SD 15.3) and 60.7% were women. People who returned the survey were on average older (50.9 years; SD 14.2) and more likely to be female (66.5%).

Of these respondents, 97% (N = 7,432) answered a question on whether they had used St John’s wort for depression, stress or worries during the past 12 months and thus form our sample and the comparison groups for this study (recent SJW users and non-SJW users). The 12 month timeframe was chosen to allow a comparison with use of health care services, self-initiated strategies used for depression, stress or worries, and other scales which measure outcomes over the same timeframe.

### Measures

In addition to the Centre for Epidemiologic Studies Depression Scale (CES-D) used to identify depressive symptoms for both the screening and outcome measures [11], the screening process included questions about the following topics and used the following validated scales:

(i) Use of health care practitioners

Participants were asked whether in the past 12 months they had seen any traditional health professionals (hospital doctor, specialist doctor, physiotherapist, psychologist, counsellor, psychiatrist, nurse, social worker, alcohol or drug worker or family therapist) or complementary therapists (chiropractor, naturopath, homeopath, acupuncturist or other natural therapist).

(ii) Strategies tried for depression, stress or worries

Participants were also asked to indicate strategies they had tried for depression, stress or worries in the past 12 months by ticking items in a comprehensive list. The list included the following activities: exercise, yoga, counselling, hypnosis, depression medication, sleeping medication, acupuncture, relaxation or meditation, massage or touch therapy, aromatherapy, changed diet, reduced use of alcohol or illicit drugs, attended self-help group for emotional wellbeing or alcohol or drug withdrawal, read a self-help book, prayer, educational or therapeutic websites, telephone helpline and talked to family or friends.

(iii) Satisfaction with general practice

The General Practice Assessment Questionnaire (Version 1.0; GPAQ) measures satisfaction with receptionists; access to practice; continuity of care; communication; practice nurses; and practice overall. The GPAQ has been validated in the UK, but also has been tested in this Australian cohort [12].

(iv) Trust

The Trust in Physician scale [13] is an eleven-item instrument presented in a five-point Likert format. The original scale was further validated and modified to make it appropriate for the primary care setting: one item, ‘*My doctor is a real expert in taking care of medical problems like mine*’ was modified to read ‘*My doctor is well qualified to manage (diagnose and treat or make an appropriate referral) medical problems like mine*’. The response labels were changed from (1 = strongly disagree; 3 = uncertain; 5 = strongly agree) to (1 = totally disagree; 3 = neutral; 5 = totally agree). The last version of the instrument was used in the *diamond* study. [14].

(v) Telephone interview

Finally, those participants who screened positive for depressive symptoms (≥16 on the CES-D) and entered the *diamond* cohort were asked at study baseline in a computer assisted telephone interview: *‘At present, who do you consider is the main person caring for your depression?’*

### Statistical analysis

Data were analysed using STATA version 11 [15] and summarised using frequencies and percentages for categorical data, and means and standard deviations (SD) for continuous data.

Participants were divided into two groups according to whether or not they reported taking SJW in the past 12 months. Logistic regression was used to examine the demographic characteristics of SJW users and to investigate the association between SJW usage and 1) health service use; 2) other strategies used for mental health care; 3) depression scores measured by CES-D; and 4) in the subgroup who screened positive for depression, whether SJW users consider themselves the main carer for their depression. Generalised estimating equations with robust standard errors were used to allow for the clustering effect, i.e. the correlation of individual responses from the same general practice.

Mixed effects linear regression models (treating the GP practice as a random effect and patient characteristics as fixed effects) were used to calculate differences in means and 95% confidence intervals (CI) for GPAQ and Trust in Physician scores. Satisfaction with nursing care was reported only for participants from clinics with a practice nurse.

A logistic regression model, used to analyse health service use and other strategies, controlled for participant characteristics including age, sex, general practice location as well as general health rating and whether they scored positively (≥16) for depressive symptoms on CES-D. These participant characteristics were chosen because of their association with patient ratings of GPs [16]. In terms of general practice location, participants were classified into Socio-Economic Indexes for Areas (SEIFA) deciles on the Index of Relative Socio-economic Disadvantage (IRSD) and Index of Relative Socio-economic Advantage and Disadvantage (IRSAD). The IRSAD provides a continuum score (or decile) of advantage (high values) to disadvantage (low values). The IRSD only measures relative disadvantage, so a high score (or decile) reflects a relative lack of disadvantage rather than relative advantage. Cut-off points of 8 in IRSD and 9 in IRSAD were created based on grouping contiguous values of SEIFA deciles in the sample into two halves, where the first half was associated with lower scores and the second half with higher scores [17].

The mixed effects linear regression models investigating associations between satisfaction, trust and various aspects of their general practice were additionally controlled for number of visits to the participant’s usual GP, and the number of GPs seen in the previous year.

The association between SJW use and caring for own depression was undertaken with logistic regression using generalised estimating equations with robust standard errors. Regression was controlled for the effects of age, sex, general practice location and additionally for depression measured by Patient Health Questionnaire (PHQ) [18].

Results are reported as unadjusted and adjusted odds ratios (ORs) with 95% confidence intervals [CI] and p values (*p*). Due to the number of analyses conducted, statistical significance was set at p < 0.01.

## Results and discussion

Of 7667 participants, 1793 (23.4%) screened positive for a probable diagnosis of depression on the CES-D (> = 16). Of participants responding to the *diamon*d screening survey question on SJW use, a total of 4.3% (320/7432; 95% CI 3.8%, 4.8%) reported using SJW in the past 12 months, compared with 18.2% (1351/7432, 95% CI 17.3%, 19.1%) who used antidepressants. The demographic characteristics of participants are shown in Table 1. SJW users were more likely to be younger, female, single, have completed higher education, employed, to have been told by their doctor that they have depression or anxiety, taking medications for depression and to see a female GP.

**Table 1:** Participant characteristics

	**Non-SJW user**	**SJW user**	**OR (95% CI)**^**†**^	***P value***
**Participant characteristics**	(N = 7112, N %)*	(N = 320, N %)*		
General practitioner location				
Urban (RRMA 1&2)	4771 (67.1)	237 (74.1)	Ref	0.20
Rural (RRMA 3-5)	2341 (32.9)	83 (25.9)	0.70 (0.41, 1.20)	
SEIFA – IRSD (8+)^‡^	3374 (47.4)	185 (57.8)	1.54 (0.99, 2.37)	0.05
SEIFA – IRSAD (9+)^§^	3410 (47.9)	188 (58.8)	1.54 (1.00, 2.37)	0.05
Age group				
18-34	1085 (15.4)	68 (21.5)	Ref	<0.001
35-54	2974 (42.2)	176 (55.5)	0.95 (0.65, 1.41)	
55-76	2982 (42.4)	73 (23.0)	0.42 (0.27, 0.64)	
Gender				
Male	2419 (34.1)	48 (15.1)	Ref	<0.001
Female	4680 (65.9)	270 (84.9)	2.71 (2.08, 3.54)	
Marital status				
Never married/Single	1235 (17.5)	89 (28.3)	Ref	<0.001
Widowed/Divorced/Separated	1245 (17.7)	70 (22.2)	0.81 (0.58, 1.13)	
Married	4559 (64.8)	156 (49.5)	0.52 (0.39, 0.69)	
Born in Australia	5735 (80.8)	268 (84.3)	1.35 (0.97, 1.88)	0.07
English is first language	6710 (94.7)	308 (96.3)	1.44 (0.76, 2.71)	0.26
Lives alone	963 (13.6)	42 (13.2)	0.94 (0.67, 1.30)	0.70
Highest level of education				
Completed year 12 or less	4038 (57.1)	133 (41.7)	Ref	0.003
Certificate/Diploma	1456 (20.6)	86 (27.0)	1.64 (1.21, 2.22)	
Bachelor degree or higher	1584 (22.4)	100 (31.3)	1.67 (1.22, 2.29)	
Employment				
Employed/Student	4504 (63.5)	229 (71.6)	Ref	0.04
Not employed^¶^	2209 (31.1)	74 (23.1)	0.70 (0.53, 0.92)	
Unable to work	383 (5.4)	17 (5.3)	0.94 (0.54, 1.65)	
Pension/benefit is main source of income	1841 (26.2)	71 (22.4)	0.88 (0.66, 1.17)	0.37
Ever told by doctor had depression	1981 (30.1)	193 (65.6)	4.22 (3.27, 5.45)	<0.001
Ever told by doctor had anxiety	1492 (24.0)	157 (56.7)	3.91 (2.96, 5.17)	<0.001
Currently taking depression medication	962 (13.7)	84 (26.3)	2.20 (1.73, 2.80)	<0.001
Long term illness/Health problem/ disability	2243 (32.3)	108 (34.5)	1.13 (0.90, 1.44)	0.29
Health rate				
Fair/Poor	1155 (16.4)	63 (19.9)	Ref	0.09
Good/Excellent	5867 (83.6)	253 (80.1)	0.78 (0.59, 1.04)	
GP female	1915 (26.9)	122 (38.1)	1.58 (1.12, 2.24)	0.009
Seen a GP 12 or more times in past 12 months	652 (9.2)	32 (10.0)	1.11 (0.75, 1.63)	0.60

OR = odds ratio. RRMA = Rural, Remote and Metropolitan Areas classification. SEIFA = Socio-Economic Indexes for Areas. IRSD = Index of Relative Socio-Economic Disadvantage. IRSAD = Index of Relative Socio-Economic Advantage/Disadvantage. *Denominators may vary due to missing data. †Odds ratios, 95% confidence intervals and *P* values with logistic regression using generalised estimating equations with robust standard errors. ^‡^Participants were classified into deciles on the IRSD and IRSAD, based on their general practitioner’s geographic location. IRSD measures relative disadvantage so a high score implies a relative lack of disadvantage. §IRSAD provides a continuum score (or decile) of advantage (high values) to disadvantage (low values). ^¶^Includes home duties, unpaid work and maternity leave.

SJW use was associated with a diagnosis of probable depression as rated by CES-D score of 16 or more (CES-D > =16: 43.8% SJW users; 23.5% non-SJW users; OR 2.44, 95% CI 1.99, 3.00, p < 0.001). We separated participants according to two recommended cut-off scores ≥16 (indicating probable depression) and ≥ 20 (indicating a significant elevation in depressive symptomatology in primary care sample). [19,20] See Table 2.

**Table 2:** Depression scores of diamond participants by CES-D score

	**Non-SJW user**	**SJW-user**	**OR (95% CI)**^**†**^	***P *** **value**
	**(N = 7112, N %)**	**(N = 320, N %)**		
Not depressed (<16)	5349 (76.5)	180 (56.3)	Ref	<0.001
16-19	473 (6.8)	36 (11.3)	2.20 (1.60, 3.03)	
20-60	1166 (16.7)	104 (32.5)	2.54 (2.03, 3.17)	

CES-D = Center for Epidemiological Studies Depression Scale. OR = odds ratio. *Denominators may vary due to missing data. †Odds ratios, 95% confidence intervals and *P* values with logistic regression using generalised estimating equations with robust standard errors.

We explored how this sample used both conventional and complementary health practitioners. After adjustment for self-rated health status, SJW users were significantly more likely to see their GP five or more times in the past year (51.3%, 43.3%), although this became non-significant when further adjusted for depression score. SJW users were significantly more likely to use all other health services, whether conventional or complementary (see Table 3).

**Table 3:** Health Service Use of diamond study participants

	**Non-SJW user**	**SJW-user**	**Unadjusted**^**†**^	***P***	**Adjusted**^**a**^^**†**^	***P***	**Adjusted**^**b**^^**†**^	***P***	**Adjusted**^**c**^^**†**^	***P***
	(N = 7112, N %)*	**(N = 320, N%)**	**OR (95% CI)**		**OR (95% CI)**		**OR (95% CI)**		**OR (95% CI)**	
≥5 Visits to GP	3072 (43.3)	164 (51.3)	1.39 (1.06, 1.82)	0.02	1.47 (1.12, 1.94)	0.006	1.39 (1.06, 1.82)	0.02	1.26 (0.97, 1.65)	0.09
Visits to	4686 (69.7)	238 (78.8)	1.46 (1.17, 1.84)	0.001	1.48 (1.19, 1.86)	0.001	1.41 (1.13, 1.77)	0.003	1.33 (1.05, 1.67)	0.02
Traditional^†^										
Visits to										
Alternative^‡^	1832 (26.6)	168 (54.0)	3.02 (2.34, 3.90)	<0.001	2.72 (2.12, 3.49)	<0.001	2.77 (2.16, 3.55)	<0.001	2.76 (2.15, 3.55)	<0.001

OR = odds ratio. GP = General Practitioner. *Denominators may vary due to missing data. †Odds ratios, 95% confidence intervals and *P* values with logistic regression using generalised estimating equations with robust standard errors.

^†^Traditional includes: hospital doctor; specialist doctor; physiotherapist; psychologist; counsellor; psychiatrist; nurse; social worker; alcohol and drug worker; family therapist. ‡Alternative includes: chiropractor; naturopath; homeopath; acupuncturist; other natural therapist.

^a^Adjusted for age, gender and general practice location.

^b^Adjusted for age, gender, general practice location and health rate.

^c^Adjusted for age, gender, general practice location, health rate and depression score.

When examining self-initiated strategies used for depression, stress or worries, SJW users were significantly more likely to have tried all listed strategies (see Table 4). For example, SJW users were more likely to have tried counsellors [adjusted Odds Ratio (adj OR) 3.45, 95% CI 2.38, 5.01] and sleeping tablets (adj OR 2.46, 95% 1.73, 3.52), but also acupuncture (adj OR 6.00, 95% CI 3.71, 9.69), hypnosis (adj OR 5.31, 95% CI 2.90, 9.74) or aromatherapy (adj OR 4.30, 95% CI 3.26, 5.67).

**Table 4:** Strategies used for depression, stress or worries

	**Non-SJW user**	**SJW-user**	**Unadjusted**^**†**^	**Adjusted**^**a**^^**†**^	**Adjusted**^**b**^^**†**^	**Adjusted**^**c**^**†**
	(N = 7112, N%)*	(N = 320, N%)*	**OR (95% CI)**	**OR (95% CI)**	**OR (95% CI)**	**OR (95% CI)**
Exercise	3207 (45.1)	256 (80.0)	3.99 (3.22, 4.95)	3.54 (2.90, 4.32)	3.51 (2.88, 4.27)	3.26 (2.63, 4.03)
Yoga	670 (9.4)	106 (33.1)	3.98 (3.09, 5.12)	3.24 (2.56, 4.09)	3.21 (2.52, 4.11)	3.03 (2.34, 3.92)
Counselling	896 (12.6)	138 (43.1)	4.75 (3.47, 6.49)	4.07 (2.93, 5.66)	3.92 (2.81, 5.48)	3.45 (2.38, 5.01)
Hypnosis	88 (1.2)	25 (7.8)	6.57 (3.53, 12.21)	5.87 (3.15, 10.93)	5.87 (3.19, 10.81)	5.31 (2.90, 9.74)
Depression medication	1222 (17.2)	129 (40.3)	3.21 (2.41, 4.26)	2.96 (2.24, 3.90)	2.81 (2.10, 3.78)	2.40 (1.71, 3.35)
Sleeping medication	828 (11.6)	96 (30.0)	3.07 (2.18, 4.32)	3.04 (2.17, 4.27)	2.89 (2.07, 4.03)	2.46 (1.73, 3.52)
Acupuncture	134 (1.9)	42 (13.1)	7.20 (4.44, 11.66)	6.62 (4.09, 10.70)	6.20 (3.86, 9.95)	6.00 (3.71, 9.69)
Relaxation/Meditation	1026 (14.4)	154 (48.1)	4.76 (3.91, 5.80)	4.09 (3.41, 4.90)	3.95 (3.27, 4.76)	3.70 (3.04, 4.50)
Massage/Touch therapy	999 (14.0)	136 (42.5)	4.05 (3.18, 5.15)	3.30 (2.66, 4.10)	3.25 (2.59, 4.08)	3.12 (2.48, 3.91)
Aromatherapy	411 (5.8)	91 (28.4)	6.16 (4.61, 8.22)	4.70 (3.59, 6.15)	4.62 (3.51, 6.09)	4.30 (3.26, 5.67)
Changed diet	1235 (17.4)	137 (42.8)	3.34 (2.70, 4.13)	2.90 (2.33, 3.62)	2.82 (2.27, 3.50)	2.59 (2.04, 3.28)
Reduced alcohol or drug	671 (9.4)	94 (29.4)	3.72 (3.02, 4.59)	3.57 (2.88, 4.42)	3.30 (2.61, 4.16)	2.95 (2.32, 3.76)
Attended self-help group						
For emotional wellbeing	158 (2.2)	31 (9.7)	4.44 (2.81, 7.00)	4.02 (2.57, 6.29)	3.49 (2.18, 5.60)	3.10 (1.94, 4.93)
For alcohol or drugs	34 (0.5)	8 (2.5)	5.45 (2.30, 12.93)	5.36 (2.18, 13.19)	4.53 (1.74, 11.76)^d^	3.96 (1.38, 11.32)^e^
Read a self-help book	921 (13.0)	163 (50.9)	6.21 (4.79, 8.07)	5.34 (4.15, 6.87)	5.17 (3.97, 6.72)	4.69 (3.54, 6.21)
Prayer	1181 (16.6)	112 (35.0)	2.72 (2.22, 3.33)	2.49 (2.02, 3.07)	2.41 (1.95, 2.99)	2.21 (1.78, 2.75)
Educational/Therapeutic websites	173 (2.4)	40 (12.5)	5.27 (3.41, 8.15)	4.41 (2.91, 6.68)	4.26 (2.78, 6.54)	3.63 (2.38, 5.54)
Telephone helpline	123 (1.7)	24 (7.5)	4.51 (2.79, 7.31)	3.55 (2.11, 5.99)	3.15 (1.81, 5.51)	2.45 (1.36, 4.42)^d^
Talked to family or friends	2724 (38.3)	218 (68.1)	3.06 (2.37, 3.97)	2.49 (1.86, 3.33)	2.40 (1.79, 3.23)	2.15 (1.56, 2.96)

OR = odds ratio. *Denominators may vary due to missing data. ^†^Odds ratios, 95% confidence intervals and *P* values with logistic regression using generalised estimating equations with robust standard errors.

^a^Adjusted for age, gender and general practice location.

^b^Adjusted for age, gender, general practice location and health rate.

^c^Adjusted for age, gender, general practice location, health rate and depression score.

All *p* values <0.001, except where indicated otherwise; ^d^*p*-value < 0.01 ^e^*p*-value < 0.1.

Table 5 shows no statistically significant differences in participants’ satisfaction with various aspects of their general practice or in their trust in their GP when adjusted for age, gender, general practice location, health rating, number of general practice visits, number of general practitioners seen and for depression score.

**Table 5:** GPAQ and Trust in Physician scores

	**Non-SJW user**	**SJW-user**										
**GPAQ item**^**‡**^	**(N = 6630)**	**(N = 277)**	**Unadjusted**^**†**^	***P***	**Adjusted**^**a**^^**†**^	***P***	**Adjusted**^**b**^^**†**^	***P***	**Adjusted**^**c**^^**†**^	***P***	**Adjusted**^**d**^^**†**^	***P***
	**Mean (SD)**	**Mean (SD)**	**COEF (95% CI)**		**COEF (95% CI)**		**COEF (95% CI)**		**COEF (95% CI)**		**COEF (95% CI)**	
**Satisfaction with:**												
Receptionists	82 (19.0)	81 (21.0)	0.26 (-1.95, 2.46)	0.82	0.36 (-1.85, 2.58)	0.75	0.38 (-1.85, 2.62)	0.74	0.12 (-2.10, 2.34)	0.92	0.35 (-1.88, 2.58)	0.76
Access to practice	68.63 (16.8)	64.8 (15.9)	-1.99 (-3.84, -0.14)	0.04	-1.65 (-3.51, 0.20)	0.08	-1.55 (-3.41, 0.32)	0.10	-1.74 (-3.59, 0.12)	0.07	-1.38 (-3.24, 0.48)	0.15
Continuity of care	77 (20.0)	75 (22.0)	-0.42 (-2.78, 1.94)	0.73	0.01 (-2.36, 2.37)	0.10	0.43 (-1.95, 2.82)	0.72	-0.003 (-2.34, 2.34)	0.10	0.22 (-2.12, 2.57)	0.85
Communication	83.96 (16.0)	85.07 (15.8)	0.44 (-1.41, 2.30)	0.64	0.04 (-1.82, 1.91)	0.97	0.17 (-1.71, 2.04)	0.86	-0.08 (-1.95, 1.78)	0.93	0.28 (-1.59, 2.14)	0.77
Nursing care§	80.03 (16.4)	77.95 (20.1)	-1.36 (-4.81, 2.08)	0.44	-1.41 (-4.87, 2.04)	0.42	-1.30 (-4.80, 2.19)	0.47	-1.27 (-4.75, 2.22)	0.48	-0.94 (-4.43, 2.55)	0.60
Practice overall	81.59 (20.4)	79.59 (19.4)	-1.45 (-3.90, 1.00)	0.25	-1.51 (-3.98, 0.96)	0.23	-1.31 (-3.78, 1.17)	0.30	-1.49 (-3.96, 0.98)	0.24	-1.10 (-3.58, 1.37)	0.38
**Trust in physician:**												
Score transformed^¥^	77.09 (13.7)	75.35 (14.7)	-1.65 (-3.29, -0.002)	0.05	-1.64 (-3.30, 0.01)	0.05	-1.46 (-3.12, 0.21)	0.09	-1.69 (-3.34, -0.04)	0.04	-1.22 (-2.87, 0.43)	0.15

COEF = coefficient. GPAQ = General Practice Assessment Questionnaire. *Denominators may vary due to missing data. ^†^COEF, 95% confidence intervals and *P* values calculated using mixed effects linear regression models (treating GP practice as a random effect and patient characteristics as fixed effects). ‡Scores are expressed as a percentage of the maximum possible score (100) for each GPAQ item, with higher scores indicating greater satisfaction. ^§^The eight general practices that did not have a practice nurse were excluded from the analysis for this item: the denominator is n = 5071 (4874/197). ^¥^Trust score transformed and weighted for missing responses.

^a^Adjusted for age, gender and general practice location.

^b^Adjusted for age, gender, general practice location and health rate.

^c^Adjusted for age, gender, general practice location, health rate, no. of GP visits and no. of GPs seen.

^d^Adjusted for age, gender, general practice location, health rate, no. of GP visits and no. of GPs seen and depression score.

Finally, those participants who fulfilled diagnostic criteria for depression on the CES-D scale were asked in a computer assisted telephone interview (CATI) (n = 717; from those there were 5 missing responses related to usage of SJW) who they considered was the main person caring for their depression (if the participant did not identify with the term ‘depression’, the interviewer then used the phrase ‘or the way you are feeling’). The association between SJW use and caring for own depression was investigated using logistic regression controlled for the effects of age, sex, general practice location and additionally for depression measured by Patient Health Questionnaire (PHQ) [18]. Half (50%; n = 32) of SJW users considered that they themselves were the main person caring for their depression, compared to 33.8% (n = 219) of non-SJW users. SJW users were more likely to consider themselves as the main person caring for their depression, and this association was significant (OR = 1.97; 95% CI 1.02, 3.83; *p* < 0.05). In contrast, only 9% of SJW users thought that a GP was the main person caring for their depression, compared to 16% of non-SJW users.

## Conclusions

### Summary of main findings

Our research has three main findings. Importantly in this large randomly selected general practice population, we found that recent SJW users were no different from non-SJW users in their satisfaction with various aspects of their general practice experience or in their trust in their GP. However, SJW users were significantly more likely to use all health services in general and to use a wide range of strategies for their depression, both conventional and complementary. Of participants currently depressed, SJW users were more likely to consider themselves the main carer for their depression, while non-SJW users were more likely to consider their GP to be the main person caring for their depression. These findings may indicate that patients who consult general practitioners and who have experienced symptoms of depression do not use SJW due to dissatisfaction with or lack of trust in their general practitioner or their clinic; rather, it may be that some aspects of their depression symptoms persist and may be more severe or less responsive to convention therapies and these participants sought additional help. We did not ask participants who it was that recommended that they try SJW, so it is possible that it was a recommendation of their GP. Regardless, patients who had recently used SJW are possibly a group more proactive in seeking care.

### Comparison with existing literature

In the *diamond* screening survey, nearly 25% of respondents met the CES-D criteria for probable depression and 18% had taken antidepressant medication in the past year. Although this is considerably higher than figures obtained by the population-based figures cited by the Australian Bureau of Statistics, who found that 9.7% of the population reported having an affective disorder [21], it is in keeping with other surveys conducted in the general practice setting [22].

Use of CAM in people with depression is common in the community. A national Finnish study found that 43% of people with ‘pure’ depression had used some type of CAM in the past 12 months, nearly always as a ‘complement’ to mainstream care [23]. We found that the use of one particular CAM, SJW, appears to be a marker of active searching for relief in people experiencing depression, not as a marker of a philosophical orientation to CAM. It is not possible from our research to explore why this is so, but this finding is supported by previous research about complementary therapies in depression [23] and generally; for example, a recent Dutch study found that many people sought complementary therapists to obtain advice from a different viewpoint, while less than 20% had concerns about their mainstream care [24].

SJW users in general practice may prefer to seek care for their depressive symptoms from a range of sources or they may prefer to take responsibility for their care – the patient as a consumer [25]. Some people prefer to avoid contact with a GP altogether [26]. Alternatively, they may seek a range of strategies as they are not obtaining the relief they need from treatment being provided by their GP, which may relate to many factors, such as concern about harm associated with pharmaceutical antidepressants, lack of response to antidepressants or more severe depression [27]. Qualitative research is needed to explore these possibilities.

The meaning of the question ‘*who do you consider the main person caring for your depression*’ is open to interpretation: it could refer to provision of therapies, to day-to-day care or to taking responsibility for choosing what kinds of therapies to use. Each of these interpretations invites a different type of response. However, it does seem that SJW users assume more personal responsibility for their depression care than non-SJW users.

### Strengths and limitations of the study

The strengths of this research include the use of a large randomly selected general practice-based sampling frame, use of validated measures of depression, satisfaction and trust, and exploration of the use of a wide range self-initiated strategies for depression, stress or worries by general practice attendees.

The limitations include a relatively low response rate and that the survey was undertaken in only one Australian state, both of which could limit generalisability, reliance on self-report and the potential for recall bias, given that participants were requested to recall information over the past 12 months. It is unlikely that recall bias would affect differentially recall about the various therapies used and therefore it should not have a major impact on the results. It is not possible to determine from this research whether use of SJW or other complementary therapies was recommended by a GP or not. The data were collected in 2005/2006; however, while this may be viewed as a limitation, popularity of complementary medicines has not diminished over this time [28], so the findings of this research remain relevant.

### Implications for future research or clinical practice

SJW use may be a marker of people searching for relief from depression and who are willing to try multiple self-help and professional treatments to deal with their problems. These people were also more likely to be users of other medicines in the past year such as antidepressants and sleeping tablets. While we cannot ascertain whether this indicates concurrent use, it certainly raises the issue of possible interactions with SJW. Authorities responsible for medicines regulation and education need to be aware of such use and put into place mechanisms to ensure that SJW users have access to information that addresses the potential risks of concurrent use.

In clinical practice, GPs should enquire about all CAM use in their patients, including SJW use, as CAM products may cause interactions or side-effects. Additionally in patients using SJW, GPs may need to enquire further about unrelieved symptoms of depression and other strategies their patients have tried; better communication about these issues may improve the doctor-patient relationship, and ultimately improve safe management of depression.

## Abbreviations

CES-D: Centre for Epidemiologic Studies Depression Scale; CAM: Complementary and alternative medicine; CATI: Computer assisted telephone interview; CI: Confidence intervals; Version 1.0 GPAQ: General practice assessment questionnaire; GP: General practitioner; IRSAD: Index of relative socio-economic advantage and disadvantage; IRSD: Index of relative socio-economic disadvantage; MHRA: Medicines Healthcare products Regulatory Agency; OR: Odds ratios; PHQ: Patient health questionnaire; SEIFA: Socio-economic indexes for areas; SWJ: St John’s wort; SD: Standard deviations; THR: Traditional herbal registration; UK: United Kingdom.

## Competing interests

The authors declare that they have no competing interests.

## Authors’ contributions

MP contributed to the conception and design of the study, interpretation of the data, drafted the article and revised it for important intellectual content. KD performed the analysis and contributed to the interpretation of the *diamond* data and revised the manuscript for important intellectual content. KF contributed to interpretation of data, and revising the manuscript for important intellectual content. MC contributed to interpretation of data, and revising the manuscript for important intellectual content. JG contributed to the conception of the study and provided access and contributed to interpretation of the *diamond* data and revised the manuscript for important intellectual content. All authors read and approved the final version of the manuscript.

## Authors’ information

MP - MS BS, M Med, FRACGP, DRANZCOG, PhD.

KD - MaSocSci, MaSci(Stat), PGDipPhil&EthStud, PGDipRelStud.

KF - BA(Hons), PgDipLaw, PgDipLegalPractice.

MC - BA, PhD.

JG - MS BS, FRACGP, DRANZCOG, PhD.

## Pre-publication history

The pre-publication history for this paper can be accessed here:

http://www.biomedcentral.com/1472-6882/14/204/prepub
